# Borderlines between Sarcopenia and Mild Late-Onset Muscle Disease

**DOI:** 10.3389/fnagi.2014.00267

**Published:** 2014-09-29

**Authors:** Johanna Palmio, Bjarne Udd

**Affiliations:** ^1^Department of Neurology, Neuromuscular Research Center, Tampere University Hospital, University of Tampere, Tampere, Finland; ^2^Department of Medical Genetics, Folkhälsan Institute of Genetics, University of Helsinki, Helsinki, Finland; ^3^Department of Neurology, Vaasa Central Hospital, Vaasa, Finland

**Keywords:** sarcopenia, myopathy, late-onset, genetic, muscle imaging

## Abstract

Numerous natural or disease-related alterations occur in different tissues of the body with advancing age. Sarcopenia is defined as age-related decrease of muscle mass and strength beginning in mid-adulthood and accelerating in people older than 60 years. Pathophysiology of sarcopenia involves both neural and muscle dependent mechanisms and is enhanced by multiple factors. Aged muscles show loss in fiber number, fiber atrophy, and gradual increase in the number of ragged red fibers and cytochrome *c* oxidase-negative fibers. Generalized loss of muscle tissue and increased amount of intramuscular fat are seen on muscle imaging. However, the degree of these changes varies greatly between individuals, and the distinction between normal age-related weakening of muscle strength and clinically significant muscle disease is not always obvious. Because some of the genetic myopathies can present at a very old age and be mild in severity, the correct diagnosis is easily missed. We highlight this difficult borderline zone between sarcopenia and muscle disease by two examples: LGMD1D and myotonic dystrophy type 2. Muscle magnetic resonance imaging (MRI) is a useful tool to help differentiate myopathies from sarcopenia and to reach the correct diagnosis also in the elderly.

## Introduction

One of the most serious consequence of aging is its effects on skeletal muscle. The aging in muscle is a complex process to which various physiological and pathological mechanisms contribute (Cruz-Jentoft et al., 2010). Sarcopenia, featuring the normal age-related changes in muscle, is defined as the slow but progressing loss of skeletal muscle mass and strength occurring with advancing age (Morley et al., 2001). A term, skeletal muscle function deficit (SMFD), has also been introduced in an attempt to better define the muscle problems in advanced age (Correa-de-Araujo and Hadley, 2014). Although sarcopenia is part of the normal aging process, it has a great impact on health and functionality, because the mobility is impaired, the risk of falls and injuries is increased, ability to perform activities of daily living is decreased, and there is an increased risk of lost independence and death (Rolland et al., 2008). Sarcopenia is common in adults over the age of 65 years and its prevalence increases with age. The prevalence varies from 5 to 13% in 60- to 70-year olds and 11–50% for the population aged 80 years or older and depends on what diagnostic methods and definitions are used (Iannuzzi-Sucich et al., 2002; Wang and Bai, 2012; Patel et al., 2013).

Muscle mass is strongly age dependent. After the age of 50, approximately 1–2% of muscle mass is expected to be lost every year and between the ages of 20 and 80 years muscle mass is reduced up to 50% (Wang and Bai, 2012). Pathophysiology of sarcopenia involves both neural- and muscle-dependent mechanisms and is enhanced by multiple factors (Cruz-Jentoft et al., 2010). Continuous age-related decrease in the number of motor neurons leads to chronic denervation of muscle tissue. This is one of the most important factors leading to the loss of muscle fibers and total muscle mass, although multiple other factors contribute, such as decreased physical activity, altered hormonal status, especially anabolic hormones, decreased total caloric and protein intake, inflammatory mediators, and factors leading to altered protein synthesis (Doherty, 2003; Ryall et al., 2008). Muscle atrophy might be induced also by increased susceptibility to apoptosis caused by mitochondrial dysfunction (Edström et al., 2007). The degree of these changes varies greatly between individuals, and an accepted definition of sarcopenia for use in clinical practice is still lacking (Cruz-Jentoft et al., 2010).

**Figure 1:**
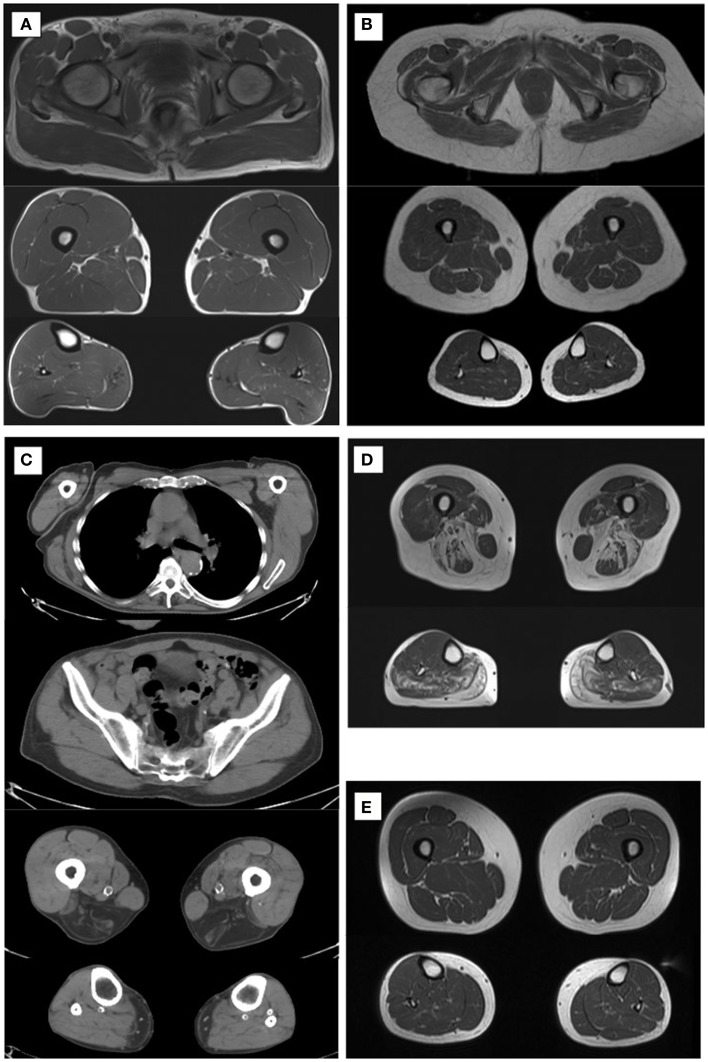
**Muscle imaging at different ages and diseases**. Normal muscle imaging in a 40-year-old male **(A)**. Normal muscle imaging in a 73-year-old male with clear decrease in muscle volume **(B)**. Slight increase of diffuse fatty tissue is seen intramuscularly. Muscle CT in a 78-year-old male with LGMD2L shows dystrophic changes in the hamstrings and gastrocnemius medialis muscles **(C)**. Muscle MRI findings in a patient with LGMD1D at age 73 with typical fatty degenerative changes in the hamstring muscles and adductors at thigh level in the soleus and gastrocnemius muscles in the calves **(D)**. Muscle MRI in a patient with DM2 **(E)** shows that muscle volume is normal, and fatty degenerative changes are not seen at age 60.

Age-related generalized loss of muscle tissue and the decrease in muscle volume and thickness are seen in muscle imaging using different techniques, such as, magnetic resonance imaging (MRI), computed tomography (CT), or ultrasonography (Figures 1A,B). Also, increased amount of intramuscular and intermuscular fat is observed (Wattjes and Fischer, 2013).

## Pathological Changes with Aging

Aged muscles show loss in fiber number and fiber size. Fiber size reduction is more prominent in type 2 fibers. The decrease in fiber number affects both fiber types but preferential loss is seen in type 2, resulting in increase in the proportion of type 1 fibers (Lexell et al., 1988). Some neurogenic changes due to motor neuron loss may be seen in muscle biopsies of very aged individuals: the “Checkerboard” appearance of the normal muscle fibers is less distinct and certain fiber grouping can be observed in aging muscle tissue. The number of hybrid fibers, expressing more than a single myosin heavy chain isoform, is also increased (Doherty, 2003). Aging gradually increases the number of ragged red fibers (RRF) and cytochrome *c* oxidase (COX)-negative fibers (Müller-Höcker, 1992). There is no clear cut-point for the amount of these changes between normal aging and mitochondrial myopathies. Thus, in cases where there are more than occasional RRF or COX-negative fibers, late-onset mitochondrial or inflammatory myopathy (inclusion body myositis) should be considered.

## Muscular Dystrophies

Usually genetic myopathies manifest at birth, in childhood, or early in adulthood, but many of them can also present at very old age (Table 1). Typically, muscular dystrophies present with slowly progressive weakness and muscle atrophy. Especially, if the symptoms are mild in severity and occur very late, the distinction between normal age-related weakening of muscle strength and clinically significant muscle disease is not always easy to make. Two entities with disease manifestations beginning at an old age are discussed in more detail.

**Table 1:** **Genetic muscle diseases that can present at a very old age**.

Muscle disease	Gene, locus	Mutation type	Age of onset
Tibial muscular dystrophy	*TTN* (titin), 2q31	Dominant, insertion–deletion, missense, truncating	35–40 distal weakness, rarely after 60 years; 60–80 proximal weakness
Welander distal myopathy	*TIA1*, 2p13	Dominant, missense	40–60 infrequently after 60 years
LGMD1A	*TTID* (myotilin), 5q31	Dominant, missense	50–60 up to 77 years
LGMD1D	*DNAJB6*, 7q36.3	Dominant, missense	20–50 up to 70 years
IBMPFD	*VCP*, 9p13.3	Dominant, missense	Mean age 42 can be over 60
Oculopharyngeal muscular dystrophy	*PAPBN1*, 14q11.2	Dominant/recessive, repeat expansion	50–60 or later in heterozygotes
DM1, late-onset oligosymptomatic	*DMPK*, 19q13.3	Dominant, repeat expansion	Over 50
DM2	*CNBP (ZNF9)*, 3q21	Dominant, repeat expansion	20–60, proximal weakness manifests later in typical cases
Acid maltase deficiency, adult-onset	*GAA* (acid α-glucosidase), 17q25.2-q25.3	Recessive, missense, nonsense, frameshift	Early adulthood up to 72 years
Mitochondrial myopathy	mtDNA defects	Dominant/recessive, maternal, sporadic, point mutations, deletions	Any age up to 70 years

*LGMD, limb-girdle muscular dystrophies; IBMPFD, inclusion body myopathy with Paget disease and frontotemporal dementia; DM1, myotonic dystrophy type 1; DM2, myotonic dystrophy type 2; mtDNA, mitochondrial DNA*.

## Limb-Girdle Muscular Dystrophies

Limb-girdle muscular dystrophies (LGMD) are a heterogeneous group of muscle diseases affecting hip and shoulder region first. There are eight autosomal dominant (LGMD1) and 23 autosomal recessive (LGMD2) genes or loci identified to date (Nigro and Savarese, 2014). Dominant diseases are usually adult-onset and milder than recessive forms, and comprise around 10% of all LGMD diseases. The age of onset of LGMD1 group of diseases is typically before the age of 50 (Nigro and Savarese, 2014) but occasional cases with later onset have been reported. LGMD1D is caused by heterozygous missense mutations in *DNAJB6* gene (Sarparanta et al., 2012). The muscle symptoms start in the proximal lower limbs at age 20–60 (Sandell et al., 2010). The weakness progresses very slowly and most of the patients remain ambulant even at very old age, although waddling gait is typical. In recessive LGMD diseases, the severity of the symptoms varies significantly. For example, LGMD2L disease caused by *ANO5* gene mutations can be very mild or even asymptomatic, especially in female patients (Penttilä et al., 2012). Of the patients examined in that study, one male patient’s symptoms started at age 70, and at first, they were considered to be a consequence of statin use. He had, however, proximal weakness in the lower limb on examination and dystrophic changes on muscle MRI (Penttilä et al., 2012). Another male patient with a very mild LGMD2L disease is now 78 years old and still walking long distances without aid. His muscle CT showed relatively severe dystrophic changes (Figure 1C).

## LGMD1D in an Elderly Patient

A 70-year-old sister, who at that time considered herself healthy, accompanied her brother to the hospital who was known to carry a *DNAJB6* mutation and who had more severe weakness and walking difficulties. She was found to carry the same mutation confirming the diagnosis of LGMD1D. At the first examination, she had mild weakness in the lower limbs, which progressed slowly during the follow-up of 10 years. Muscle MRI showed, nonetheless, dystrophic changes in her muscles compatible with the earlier findings of this disease (Figure 1D) (Sandell et al., 2013).

## Myotonic Dystrophy Type 2

Myotonic dystrophies are the most common forms of muscular dystrophies in adults, but exact epidemiological data for myotonic dystrophy type 2 (DM2) are lacking. DM2 is, however, quite common at least in European populations. The mutation frequency is 1 in 1830 in the Finnish population, suggesting a clinical manifestation frequency of 1 in 5000 (Suominen et al., 2011). Dominantly inherited DM2 results from a (CCTG)n expansion in CNBP gene (formerly ZNF9 gene) (Liquori et al., 2001). The phenotype of DM2 is highly variable in severity, characterized by adult- or late-onset proximal muscle weakness, myalgia, and myotonia. Beyond the skeletal muscle symptoms, it is a multi-system disease and can cause cardiac conduction deficits, cataracts, and hormonal problems, such as insulin resistance, mild cerebral involvement, and liver enzyme elevation (Machuca-Tzili et al., 2005; Udd and Krahe, 2012).

## DM2 in Elderly Patients

A 64-year-old female has had a slowly progressing myalgic syndrome for 15 years. The investigations begun when she experienced recurrent rhabdomyolysis at age 56 while on statin medication. Electromyogram showed spontaneous activity high frequency discharges and increased insertional activity but no myotonia. Between rhabdomyolysis periods, the levels of creatine kinase (CK) were normal. Myalgia reduced her physical performance; she was only able to walk 100 m at normal pace. Otherwise, the muscle strength was normal and muscle MRI was within normal limits (Figure 1E). Muscle biopsy showed minor changes suggestive of DM2: a subpopulation of highly atrophic type 2 fibers, nuclear clump fibers, and increased amount of internal nuclei. DNA analysis revealed (CCTG)n expansion in *CNBP* gene confirming the diagnosis of DM2. The mutation was transmitted by her mother, now at age 90. The mother was investigated after the diagnosis was made in the daughter and proved to have had some progressive walking difficulties and used a stick for walking since age 80. She had regular visits at the primary health care because of type 2 diabetes but her muscle weakness had not been noted as abnormal for age. At examination, she had moderate proximal lower limb weakness, even though she thought herself healthy for the age.

## Discussion

Muscle weakness, gradually worsening walking difficulties, muscle pain, and stiffness are common complaints in the elderly. Sarcopenia begins in mid-late adulthood, but the age of onset and the rate of muscle loss vary greatly between individuals. Normal age-related processes lead to certain findings in muscle histology and imaging studies. It is not always self-evident to consider the possibility of a late-onset myopathy as a cause of muscle symptoms in elderly people. However, old age should not limit investigations.

Comprehensive studies on myopathies in the elderly are scarce. Laguno et al. (2002) studied muscle biopsies from 239 patients over 65 years and diagnosed specific myopathies in 36% of them. Most of the causes of myopathies were other than genetic, i.e., inflammatory myopathies and vasculitis being the most frequent. They identified dystrophies or congenital myopathies in 9% and metabolic myopathies in 10% of the patients. Elderly patients showed more non-specific type 2 fiber atrophy and fewer normal muscle biopsies compared to younger patients (Laguno et al., 2002). This was also shown in a similar study by Lacomis et al. (1993). These studies proved that muscle biopsies are useful also in the elderly. The findings can be subtle or non-specific as in our patient with DM2, although together with the myalgic syndrome, they were suggestive of DM2.

DM2 is a heterogeneous disease, and in the mild end of the spectrum, not always obvious to suspect. This is particularly true for patients without myotonia even on EMG and with normal CK levels, as was the case in the DM2 patient and in the LGMD1D patient examples. Electromyogram is usually myopathic in muscular dystrophies but can be normal and without myotonia in DM2 (Udd and Krahe, 2012). Many DM2 patients experience myalgia, typically induced by exercise, as initial manifestation. The myalgic syndrome in DM2 cannot be distinguished from fibromyalgia (Udd and Krahe, 2012). Proximal muscle weakness manifests later, usually after 50–60 years of age, even though, patients can remain asymptomatic until old age. Rhabdomyolysis is not a known manifestation of DM2, and without rhabdomyolysis, our patient could still be undiagnosed. She has probably a second unknown cause for rhabdomyolysis. Otherwise, the muscle disease was quite mild and even milder in her mother showing the milder end of symptom severity of DM2.

Muscle MRI (or CT) is useful and currently a widely used tool to assess the distribution of affected muscles and to aid in obtaining targeted muscle biopsy. Dystrophic changes, i.e., fatty degenerative changes in muscle, are more reliably detected on MRI compared to clinical evaluation. The pattern of affected muscles can direct the diagnostic genetic investigations, which is explicit in the case of LGMD1D (Sandell et al., 2013). When evaluating a patient with a possibility of a neuromuscular disorder, a concomitant neurogenic disease is more prevalent in the elderly, such as neuropathy or degeneration of the lumbar vertebrae causing radiculopathy. Neurogenic and myogenic changes can, to some extent, be distinguished by muscle imaging and sometimes unnecessary investigations or even a surgical operation can be avoided by correct diagnosis.

The specific cause of the symptoms is the key to assess the right therapeutic and rehabilitation measures, and to estimate prognosis. A possibility of a genetic myopathy is important to bear in mind also in the patients with very late-onset symptoms. If a genetic muscle disease is diagnosed, it can have an impact for the wider family and genetic counseling can be applied. Muscle MRI is useful in the distinction and a recommended tool to help differentiate myopathies from sarcopenia and to reach the correct diagnosis also in the elderly.

## Author Contributions

Dr. Johanna Palmio: substantial contribution to the design of the work, drafting the work, final approval of the version to be published, and agreement to be accountable for all aspects of the work in ensuring that questions related to the accuracy or integrity of any part of the work are appropriately investigated and resolved. Dr. Bjarne Udd: substantial contribution to the design of the work, revising the work critically for important intellectual content, final approval of the version to be published, and agreement to be accountable for all aspects of the work in ensuring that questions related to the accuracy or integrity of any part of the work are appropriately investigated and resolved.

## Conflict of Interest Statement

The authors declare that the research was conducted in the absence of any commercial or financial relationships that could be construed as a potential conflict of interest.
